# Genome-wide analysis of Alu editability

**DOI:** 10.1093/nar/gku414

**Published:** 2014-05-14

**Authors:** Lily Bazak, Erez Y. Levanon, Eli Eisenberg

**Affiliations:** 1 Mina and Everard Goodman Faculty of Life Sciences, Bar-Ilan University, Ramat Gan 52900, Israel; 2 Raymond and Beverly Sackler School of Physics and Astronomy and Sagol School of Neuroscience, Tel Aviv University, Tel Aviv 69978, Israel

## Abstract

A-to-I RNA editing is apparently the most abundant post-transcriptional modification in primates. Virtually all editing sites reside within the repetitive *Alu* SINEs. *Alu* sequences are the dominant repeats in the human genome and thus are likely to pair with neighboring reversely oriented repeats and form double-stranded RNA structures that are bound by ADAR enzymes. Editing levels vary considerably between different adenosine sites within *Alu* repeats. Part of the variability has been explained by local sequence and structural motifs. Here, we focus on global characteristics that affect the editability at the *Alu* level. We use large RNA-seq data sets to analyze the editing levels in 203 798 *Alu* repeats residing within human genes. The most important factor affecting *Alu* editability is its distance to the closest reversely oriented neighbor—average editability decays exponentially with this distance, with a typical distance of ∼800 bp. This effect alone accounts for 28% of the total variance in editability. In addition, the number of *Alu* repeats of the same and reverse strand in the genomic vicinity, the expressed strand of the *Alu*, *Alu*’s length and subfamily and the occurrence of reversely oriented neighbor in the same intron\exon all contribute, to a lesser extent, to the *Alu* editability.

## INTRODUCTION

Post-transcriptional modifications of mRNA are very common. Only a few of these are well characterized, as they are directly detectable by cDNA sequencing. In particular, adenosine deamination into an inosine (A-to-I RNA editing), catalyzed by enzymes of the ADAR (adenosine deaminases that Act on RNA) family, has been extensively studied in the past decade (1–4). Millions of adenosines in the human transcriptome can undergo A-to-I editing (5–9) almost all of them are adenosines within *Alu* repeats (10–13) virtually all of which are subject to A-to-I editing (9).

The *Alu* SINE (short interspersed nuclear element) is the most abundant primate-specific retro-transposon, ∼300 nucleotides in length (14–16). *Alus* make up more than 10% of the human genome mass, with some 1.195 million copies (UCSC browser, hg19 genome version), an exceptionally high number for a single SINE.

Many Alu repeats are embedded within genes, and are thus transcribed as part of the pre-mRNA transcription of the gene by pol-II. Due to the high copy number, it is likely that an *Alu* and a counterpart, oppositely oriented, *Alu* exist nearby and are transcribed together within the same mRNA molecule. As the mRNA transcript folds, these two *Alus* may form RNA secondary structures that are targeted by the double-stranded RNA (dsRNA) binding ADARs (17).

A-to-I editing in *Alu* exhibits a puzzling specificity and selectivity in the adenosines which are edited. For example, the E1 site within an *Alu* repeat in the NARF gene (18) is edited extremely efficiently, with nearly 100% of transcripts showing an inosine. In the generic *Alu* element, one observes a seemingly random editing pattern with a highly varying editing level across the adenosines within the repeat.

However, this pattern is remarkably consistent across different individuals (19). Sequence and structural motifs that affect the editing levels of specific adenosines have been previously documented (20–23). These motifs are too weak, however, to fully explain the variability in A-to-I editing efficiency. Therefore, the question still stands: what controls the editing level at each given site? Moreover, it is well known that editing within repeats exhibits itself in clusters of edited sites. This correlation between different adenosines within the same repeats suggests that there are parameters characterizing the whole *Alu* repeat which affect its editability, beyond the local site-specific structural and sequence motifs.

Here, we focus on these global parameters and look for the different characteristics that determine the editing level of the whole *Alu* element. Previous studies have pointed out to several features that are associated with edited *Alu* repeats, such as the existence of a nearby reversely oriented *Alu*, and the distance to it (10–13). A recent genome-wide study (9), based on two large RNA-seq data sets, provides us with a genome-wide map of editing levels for the *Alu* elements in the human genome to an unprecedented accuracy. Using these data, we are able to quantify the different determinants, some of which already suggested and others that have not been yet described.

Our main findings are (i) the distance to the nearest reversely oriented *Alu* is a critical parameter—editing of the *Alu* repeat decays exponentially, on average, with this distance, with a typical length scale of ∼800 bp. This parameter alone accounts for 28% of the variability in editing levels across *Alu* repeats, (ii) editing levels are positively correlated with the number of reversely oriented repeats in the genomic vicinity of the *Alu*, and negatively correlated with the number of same-strand repeats, (iii) editing is stronger in *Alus* whose length is close to the typical value and when the closest reversely oriented *Alu* is long and resides in the same intron/exon, (iv) the consensus strand of the *Alu* repeats is more strongly edited than the reverse strand, and show a slightly different distance dependence.

## MATERIALS AND METHODS

### RNA-seq data

Two large RNA-seq data sets were used: Human BodyMap 2.0 Project (GEO accession number GSE30611, HBM) that consists of RNA-seq of 16 human tissue types and a second RNA-seq data set (Sequence Read Archive accession SRA043767.1, YH) (6), that was derived from a lymphoblastoid cell line of a male Han Chinese individual (YH). (See (9) for more details.)

### Statistical analyses

**Figure 1.**
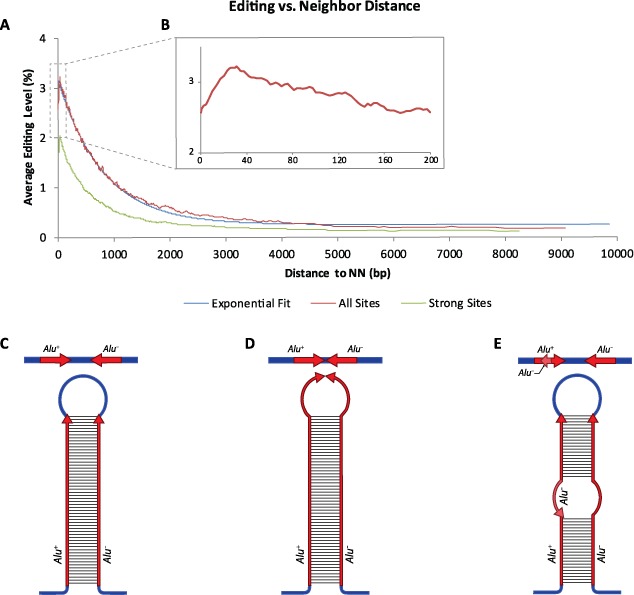
Distribution of *Alu* editibility. Editability is calculated as the ratio of inosines to (inosines+adenosines) in all reads coming from the *Alu* element of choice (see (9) for more details). Almost all *Alu* elements are edited to some extent, but editability is typically less than 1%.

The effect of the various parameters tested on editability was assessed by directly observing the correlation of the two in the available data, as presented in the figures. Based on these observations, linear models was used (with the exception of the distance dependence, which is very well described by an exponential fit, see Figure 1) and standard ANOVA methods were implemented to assess the importance of each specific effect on editability. Only the distance of the nearest reversely oriented repeat was found to explain a sizable fraction (28%) of the total variance. Nonlinear curve fitting was done using the Grace plotting tool.

## RESULTS

### Alu editability

It has been pointed out long ago that editability correlates with several structural genomic factors, including the distance to a neighboring reversely oriented *Alu* (10–13). These finding are consistent with our understating of the dsRNA structure as a pre-requisite for editing (24). However, they were based on low-coverage expression data (typically few million reads altogether), which resulted in low sensitivity of the editing detection algorithms, and very poor accuracy in determining the editing levels per site in a genome-wide fashion (9).

The next generation sequencing era have opened new directions in expression quantification, and revolutionized editing detection as well (5–7,25,26). In a recent paper (9), we have analyzed two large RNA-seq data sets and obtained a genome-wide quantification of the editing levels for all *Alu* elements in the human genome. Importantly, we found that virtually all *Alu* elements are being edited to some extent, thus making the past distinction between ‘edited’ and ‘non-edited’ elements obsolete. Instead, one should look at the editing level, which varies considerably (up to three orders of magnitude) across individual adenosine sites. As most sites are edited to a low level, less than 1% typically (9), one would need to cover the whole transcriptome with >>1000 reads in order to determine accurately the editing level in each site, per sample. This kind of coverage is still beyond the capabilities of a single experiment using current sequencing technology, and certainly is not provided by available data sets.

Secondary structure is expected to be a major component determining editability (17,20,24,27), and is, by nature, a property of the whole *Alu* repeat or large parts of it. Thus, there is a reason to expect *a priori* a global effect affecting the overall editing level of a certain *Alu* repeat beyond the local sequence and structural motifs affecting editing levels of each site. This hypothesis is indeed supported by many previous studies, reporting positive correlation in editing levels between adenosines belonging to the same *Alu* repeat (10–13). We therefore focus here on this aspect of editability, and try to pinpoint the features of an *Alu* element that make it more or less edited as a whole.

Using the data from the above work, we calculated for each *Alu* element the average editing level per adenosine included in the element, that is, the ratio of inosines to (inosines+adenosines) in all reads coming from the *Alu* element of choice (see (9) for more details). As this quantity is an average over thousands of adenosines, typically, it is much more robust than the individual editing levels in each site. We limited ourselves to highly covered *Alu* sequences: (i) we considered only those genomic Alu sequences for which at least 30 adenines along the genomic Alu (out of all adenines in a typical Alu, 63 in average) were covered, (ii) we counted the number of adenosines/inosines found in all reads aligned to these genomic locations combined, and required that a total of at least 1000 adenosines (or inosines) were sequenced. For a general *Alu* repeat, it is not known which strand is expressed (and indeed, we have provided evidence (9) that both strands are expressed to some level (28,29)). We thus chose to focus on the 203 798 *Alu* repeats that reside within RefSeq genes, and assumed all reads mapped to these elements to have come from the RefSeq strand. This assumption is correct for >90% of the reads, see (9) for more details. Note that our data set did not include hyper-edited reads that were not mapped uambiguously to the *Alu* repeat (8). Thus, our editing level might be underestimated for heavily edited targets. The average editability (fraction of adenosines converted to inosines) of an *Alu* element within a RefSeq gene is 1.34 (%), with a standard deviation of 1.72 (see the full distribution in Figure 1).

### Distance to the closest reversely oriented Alu

**Figure 2.**
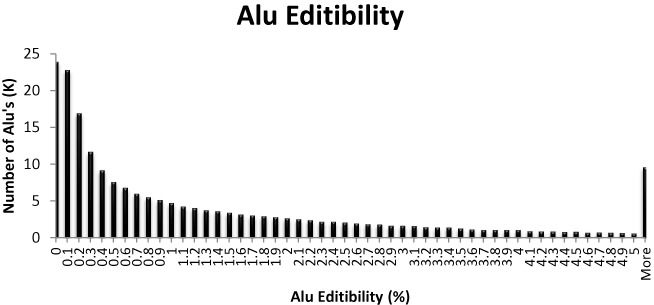
Editing level increases with decreasing distance to the nearest reversely oriented neighbor. (a) The distance dependence closely follows an exponential function, with a typical decay distance of ∼800 bp (see Equation (1)). The same trend is observed when looking only at strong editing sites, where the editing level is >25%. (b) A slight decrease in editability is observed for *Alu* elements which are very close to their reverse neighbor. (c–e) Too small a distance is detrimental for editing. A schematic illustration of two reversely oriented neighboring *Alu* elements that form a dsRNA structure. (c) A long dsRNA structure is formed when both *Alu* elements has an optimal distance between them. (d) When the two *Alu* elements are too close, with no spacing between them, the RNA flexural rigidity prevents full pairing. In this case, the RNA bases next to the neighboring ends of the *Alu* elements are likely to be less tightly bound, and thus less edited. (e) A case of nested *Alu* elements. A positive strand *Alu* resides in the middle of a negative strand *Alu*. Here too, although the distance between the elements is formally zero, pairing is negatively affected.

Having found the editability for each *Alu*, we looked at the dependence on the distance to the nearest neighbor *Alu*. As expected, we see stronger editing for elements with a closer neighbor. Previous works have usually provided only cutoff values for the distance, i.e. editing is possible if the distance is lower than some value, usually 2000–3500 bp (10–13,30). Interestingly, we find that the distance dependence closely follows an exponential function (Figure 2), with a typical decay distance of ∼800 bp:

(1) \begin{equation*} {\rm E} = 2.9 \cdot e^{ - d/800} + 0.25 \end{equation*}

where E is the editability in percent and *d* is the distance to the nearest reversely oriented *Alu* repeats (in bp). The value of 0.25% editability fitted for large distances is mostly due to the base-line false-positive error level associated with the sequencing and our detection algorithm. In order to show that, we also present the data as obtained when one looks only at strong editing sites, where the editing level is >25%. Indeed, in this data set the average signal is very close to zero for large distances between the neighboring *Alus*. Thus, one may conclude that the average editability of an *Alu* element with a neighbor 2400 bp apart is ∼20 times lower than that of an *Alu* repeat with a close neighbor (say 100 bp apart).

An interesting exception to the above rule is the slight decrease in editability observed for *Alu* elements which are very close to their reverse neighbor (see Figure 2b). This can be explained as follows: generally, the double-strand structure is stronger the closer the neighbor is. However, flexural rigidity of RNA and the associated proteins bound to it may disfavor full pairing when the two *Alu* elements have no spacing between them. In this case, the RNA bases next to the neighboring ends of the *Alu* elements are likely to be less tightly bound, and thus less edited (Figure 2d). Moreover, in many cases *Alu* elements are nested, e.g. one positive strand *Alu* resides in the middle of a negative strand *Alu*. Such cases have zero distance between the elements, but again pairing is negatively affected (Figure 2e).

The distance to the neighboring *Alu* repeat alone explains 28% of the total variance in editability of *Alu* elements.

### Number of Alu elements in the genomic neighborhood

**Figure 3.**
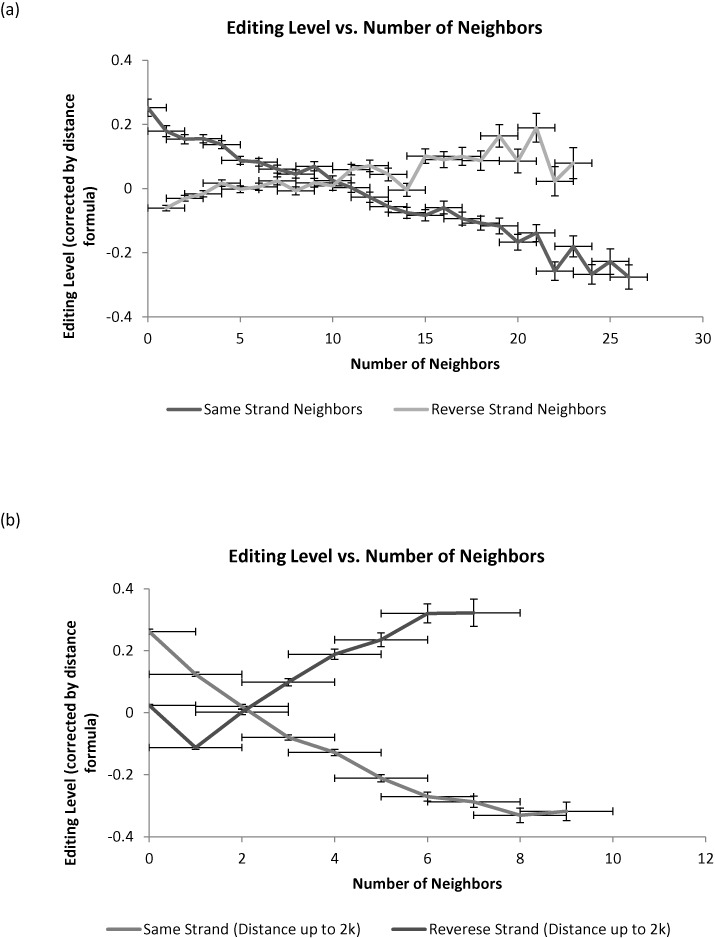
Number of *Alus* in the neighborhood affects editability. (a) One finds a positive correlation of editability with the number of reversely oriented repeats in the genomic neighborhood (10 000 bp each side) and a negative correlation with the number of same strand elements. (b) The effect is even stronger when looking at the immediate neighborhood (2000 bp each side). Note that we plot the difference between the observed editing level and the average level for all Alu whose nearest neighbor is at the same distance (formula (1)). This difference could be positive or negative.

It was already claimed that having many reversely oriented elements nearby increases the probability of an *Alu* element to be edited (10–13,30,31). In addition, we hypothesized that having many neighbors of the same orientation should reduce editability, as these same-orientation neighbors compete with the *Alu* of choice and reduce its probability to bind and form a dsRNA. There is a very strong correlation between the density of reversely oriented elements in the vicinity of an *Alu* to the distance to the closest neighbor—the more neighbors there are, the more likely is one of them to be very close. Thus, in order to properly examine the above two hypotheses one needs to control for the effect of the distance to the nearest neighbor. Given the above functional form (1), one can find the residual (positive or negative) editability of an *Alu* element beyond what is expected on average based on its distance to the nearest neighbor. This residual editability may then be correlated to the number of reversely oriented, or same strand, elements in the genomic neighborhood (10 000 bp each side). Indeed, one observes a positive correlation of editability with the number of reversely oriented neighbors, and a negative correlation with the number of same strand ones (Figure 3). The effect is even stronger when looking at the immediate neighborhood (2000 bp each side, Figure 3b).

Note that these two latter (opposing) effects are also interfering—the existence of many same-strand *Alus* in a region is positively correlated with the existence of many reversely oriented *Alu* there. In order to separate these two effects, and given the linear trend seen in Figure 3, we have used linear regression analysis to obtain the combined result of the two effects, leading to:

(2) \begin{equation*} {\rm E} = 2.9 \cdot e^{ - d/800} - 0.022{\rm Nss} + 0.015{\rm Nrs} + 0.36 \end{equation*}

where Nss is the number of same-strand *Alu* neighboring repeats and Nrs is the number of reversely oriented neighboring repeats. E is the editability in percent, *d* is the distance to the nearest. For example, the existence of 10 same-strand elements within 10 kbp of an *Alu* reduces its editability, on average, by 0.22%, while existence of 10 reversely oriented elements increases its editability, on average, by 0.15%. This effect is roughly linear in the number of neighbors.

### Alu length

**Figure 4.**
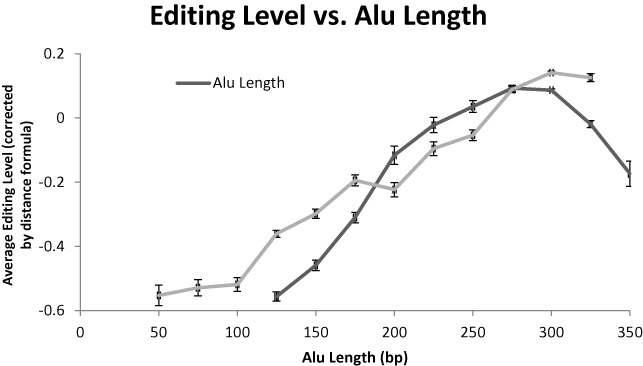
Editibility versus *Alu* length. *Alus* much shorter than the typical length are less editable, as they form weaker dsRNA. Elements too long are also less edited, on average, since their neighbor can bind to only a part of the long element. The longer the neighboring repeat is, the stronger the editing.

It is important to stress that we define editability as the fraction of adenosines being converted. Thus, having less adenosines and less putative editing sites should not affect editability naively. However, we do see that shorter *Alus* are less editable (Figure 4). This is probably due to the weaker dsRNA structure created by these shorter elements. In addition, very long *Alu* elements, longer than the typical value of ∼300 bp, are also less well edited, as the dsRNA structure that forms upon binding to their neighboring *Alu* is unlikely to cover them fully. Similarly, having a short *Alu* as a reversely oriented neighboring *Alu* also reduces editability for the same reason. Altogether, these effects explain only ∼4% of the remaining variance.

### Intron–exon structure

**Figure 5.**
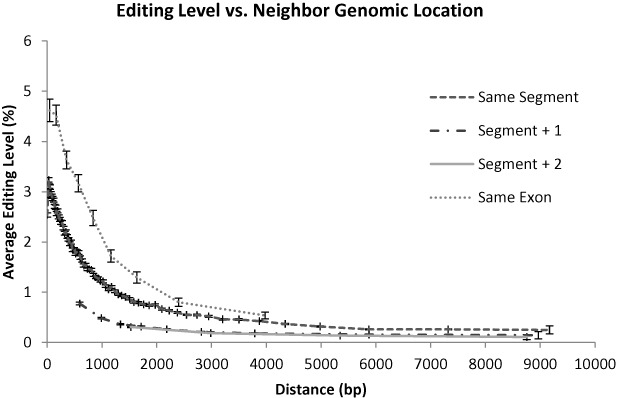
Pairing with a repeat in the same exon facilitates stronger editing. Editing is weaker when the closest neighbor resides in a different gene segment (exon\intron). Elements in an exon, with a nearest neighbor on the same exon, are very highly edited.

RNA editing is known to occur in concurrence with splicing (32). Thus, if one of the pairing *Alu* elements resides in an intron, editing could be suppressed once the intron is spliced. In fact, it was shown long ago that editing due to pairing (33) with a different RNA molecule (e.g. antisense transcripts) is not favorable (34,35). Accordingly, we checked whether having the two *Alu* elements on the same intron (or exon) affects their editability. Again, being on the same intron\exon could facilitate editing only due to making the distance shorter. We have, therefore, compared the editability as a function of distance (Figure 5). Indeed, average editing is weaker when the closest neighbor resides in a different gene segment (exon\intron). Elements in an exon, with a nearest neighbor on the same exon, are much more edited (Figure 5). This could be due to the possibility of their editing even after splicing, in the nucleus or maybe even in the cytoplasm.

However, note that while these two additional effects are significant, they do not contribute much to explaining the variance between individual elements.

### Alu strand dependence

The two strands of the *Alu* repeat are very different in terms of editing. The positive strand of the consensus sequence includes two poly-A regions which are a preferred target for ADARs (8), while elements transcribed from the reverse complement to the *Alu* consensus strand contain two poly-U regions, not editable obviously. The average number of adenosines per *Alu* element is 83.8 for the consensus (plus) strand and 52.7 for the minus strand. This explains why the total editing signal is much stronger for elements transcribed from the plus strand (8).

**Figure 6.**
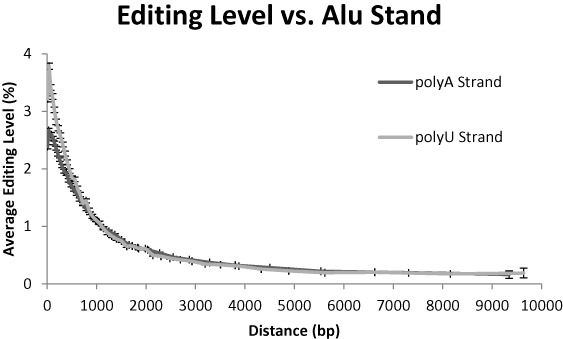
Editing versus *Alu* strand. Poly-A strand is more editable. Moreover, the distance dependence is different between the two strands. Up to a distance of ∼700 bp poly-U *Alu* elements are significantly more editable, while the reverse is seen when the neighbor further apart.

However, here we look at the inosines fraction, or editing per adenosine. We did not expect *a priori* any difference between the two strands. Surprisingly, they behave quite differently (Figure 6). On average, poly-A strand is more editable, however, there is a difference between elements with a very close neighbor, up to ∼700 bp, and other elements. In the former, poly-U strand elements are significantly more editable, while in the latter the difference is barely detectable. We have no convincing explanation for this surprising finding.

The distance dependence of the editability in the two strands is well approximated by the following exponential functions:

(3) \begin{equation*} \begin{array}{*{20}c} {{\rm poly-A}:\quad {\rm E} = 2.46 \cdot e^{ - d/960} + 0.25} \\ {{\rm poly-U}:\quad {\rm E} = 3.33 \cdot e^{ - d/710} + 0.29} \\ \end{array} \end{equation*}

The two strands vary significantly in their variance too. For poly-A, the nominal standard deviation is 1.48 (reduced to 1.24 after controlling for the distance), compared to 1.91 for poly-U (1.61 after controlling for the distance).

### Similarity to the neighboring Alu

**Figure 7.**
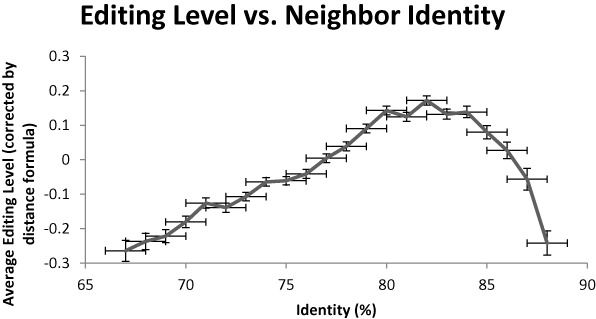
Editing versus similarity between the two neighbors. We used BLAST alignment as a proxy for sequence similarity. The more similar the *Alu* repeats are, the higher is the editing level. Pairs with extremely high identity score are less well edited. Possibly, this is due to the almost perfect dsRNA helix formed, lacking of A:C pairing in the secondary structure, which are preferred targeted by ADARs.

We explored the influence of the similarity of an *Alu* to its neighbor on the editing level, looking at a parameter that characterizes the binding between the two neighboring *Alu* repeats. Full calculation of the secondary structure for hundred thousands pairs is time consuming, and we therefore used the identity between the two neighbors, given by a simple Basic Local Alignment Search Tool (BLAST) alignment, as a proxy for their similarity (Figure 7). As expected, the more similar the *Alu* repeats are, the higher is the editing level, but this effect is rather weak, accounting for an insignificant part of the variance. Interestingly, pairs with extremely high identity score are less well edited. One may argue that this results from the correlation between Alu length and its identity to the neighbor, as Alus with very high similarity to their neighbor tend to be somewhat shorter, on average. Yet, even upon limiting the analysis to normal length Alus (270–300 bp), the downward trend in editability for high-identity Alu pairs persists. Possibly, this is due to the almost perfect dsRNA helix formed, lacking of A:C pairing in the secondary structure, which are preferred targeted by ADARs (10).

We have also checked parameters characterizing the similarity of the *Alu* repeat to its consensus sequence, such as the Smith–Waterman score, *Alu* diversity, deletions and insertions. The trends observed were all in concordance with expectations (data not shown), but did not show any meaningful contribution for explaining editability variance.

### Alu families

*Alu* elements are divided into several subfamilies (*AluJ*, *AluS*, *AluY* and the single-armed, shorter, *FLAM*, we have used Repeatmasker annotation as it appears in UCSC). We find that having the nearest neighbor *Alu* of the same subfamily has a positive effect on editability (44% of the repeats have a nearest neighbor of the same subfamily, their averaged editing level is 1.44%, compared to 1.29% for other *Alu* repeats). Interestingly, having the nearest neighbor of the same subfamily does not further improve editability (only 7.5% of the edited *Alu* have a nearest neighbor that belongs to the same subfamily, their averaged editing level is 1.39%, lower than the average for same subfamily). Pairs of *Alu* repeats from the same subfamily are, on average, slightly further apart. However, even after correcting for distance, using Equation (1), we find that having a neighbor of the same subfamily, but not of the same subfamily, increases average editabilty by +0.23% as compared to different families, while having a neighbor of the same subfamily increases average editabilty only by +0.19% as compared to different families. This surprising (although minor) effect might be related to the lowered editability in highly similar neighbors (Figure 7).

**Figure 8.**
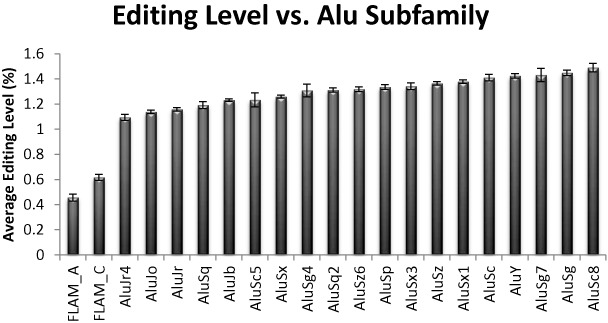
Editing in the various *Alu* subfamilies. Similar picture is observed after correcting for distance to the nearest reversely oriented *Alu*.

Overall, editing of *AluJ* elements is weaker (average editing level 1.18; *AluS*: 1.34 and *AluY*: 1.40 and *FLAM* only 0.56). These numbers correlate to the diversity within these families, and their age. Further differences are seen among the various subfamilies (Figure 8). Again, these difference account to only ∼1% of the remaining variance.

## DISCUSSION

It has been recently shown that virtually all *Alu* repeats in the human genome undergo A-to-I editing (9). However, the editing rate varies dramatically between individual *Alu* sequences. In this work, we have studied, for the first time, the variability in editing levels, and shown that a large fraction of the variance in editability of specific *Alu* repeats can be accounted for by looking at the genomic structure of the *Alu* sequence and its vicinity. In particular, we find that the distance to the nearest neighbor has a major effect on editability, much stronger than all other determinants studied. These results can be of use for any future study aiming at detecting Alu editing, editing in repeats in other organisms, as well as studies looking for editing in close vicinity to edited paired Alu repeats (36).

In principle, one would have wanted to have a full predictive model, enabling a prediction of the editing level (per site or per *Alu* sequence). Yet, all of the effects mentioned in this work account for only ∼1/3 of the total variance observed in the HBM and YH data sets. The question then arises what explains the remaining variance?

One factor which was studied extensively is the local sequence motif. ADAR (or possibly other RNA binding proteins that affect editing levels) has a specific sequence preference (20). Variations between individual *Alu* sequences cause differences in the compatibility of the many adenosines in them to the preferred local sequence motif, e.g. a single nucleotide change in a specific *Alu* that removes a ‘G’ upstream to an adenosine in a given *Alu* repeat could dramatically enhance editability of that specific site (10,23). In addition, local variations might affect the double-stranded structure required for editing. For example, a single nucleotide change that results in an A:C mismatch in the dsRNA formed could enhance editability. On the other hand, short insertions or deletions could introduce bulges in the secondary structure which are detrimental for editing.

**Figure 9.**
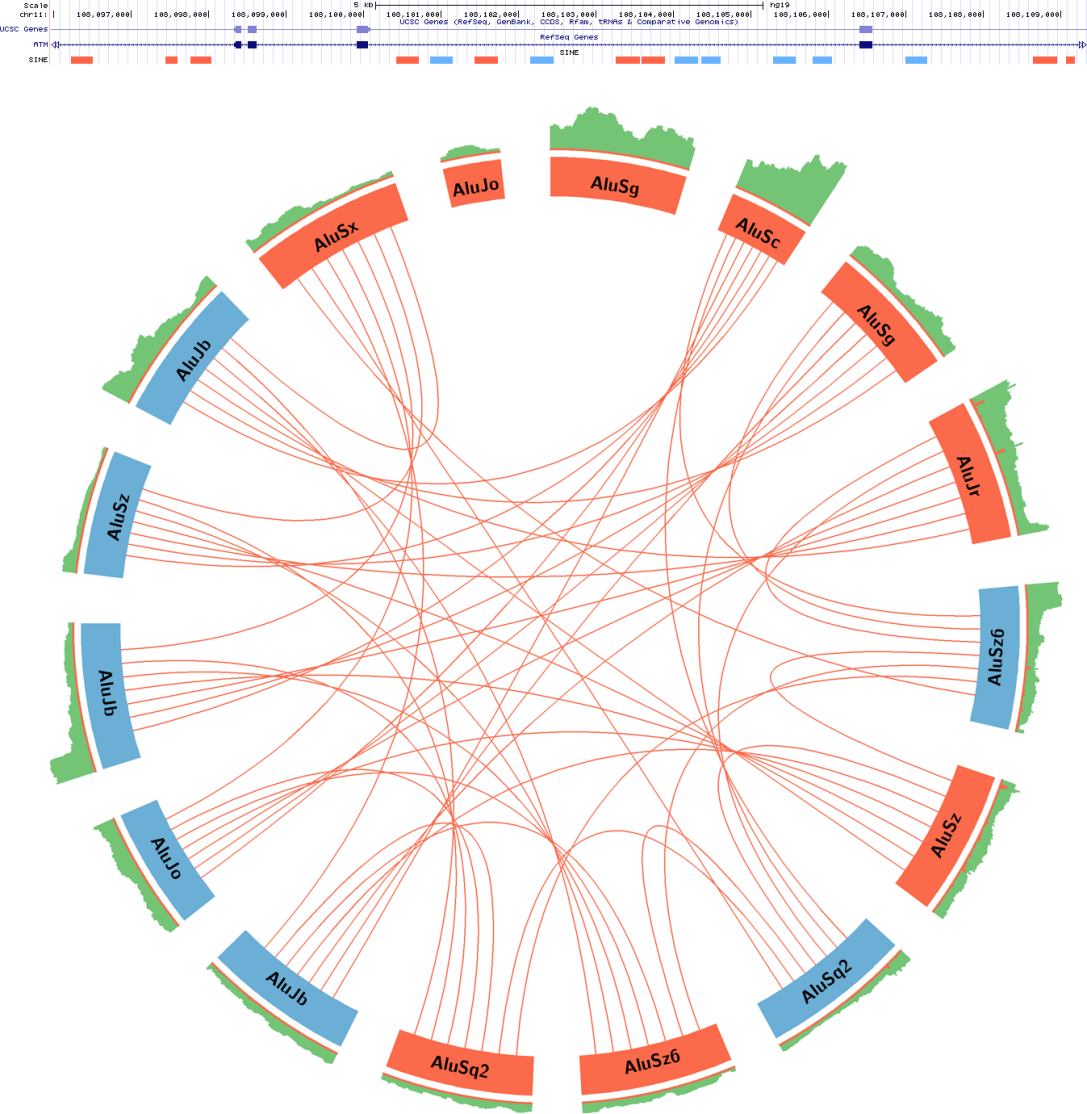
A typical genomic 10k bp neighborhood of an *Alu*. The UCSC track presents a part of the *ATM* gene, which shows four of its exons. This part of the gene includes 16 intronic *Alus*, 9 are in ‘+’ orientation and 7 are in the ‘-’ orientation. Below, appears a figurative representation of the putative paired-Alu dsRNA structures that might form. *Alus* are shown around the outer ring and are oriented in a clockwise direction with ‘+’ *Alu* indicated in red and ‘–’ in pale blue. Neighboring inverted non-diverse *Alu* closer than 3500 bp are connected by a line. Other tracks contain coverage (light-green bars) and editing levels (light-orange bars). Two of the *Alus* are not editable according to our criteria.

Another important factor that might contribute to editing variability is the structure of the *Alu* neighborhood. In this work, we have only referred to the nearest reversely oriented *Alu* repeat. However, the full picture is much more complex. A typical *Alu* repeat has many neighboring *Alu*'s and the folding of the entire transcript should be considered (see Figure 9). In addition, editing itself might modify the RNA folds, so the relevant paired *Alu* element might change dynamically.

One also has to bear in mind the tissue variability. Our data set is composed of RNA-seq of 16 different tissues. It is well known that editing level vary across tissues (37). Thus, *Alu* sequences within a gene that is more widely expressed in tissues with higher editing levels will appear as more editable that *Alu* sequences that are mainly expressed in less well-edited tissues. Current amount of available data still does not allow us to go into a single-tissue resolution while keeping the high coverage we need. In the future, it will be interesting to dissect the effects of tissue expression profile from the editability in any given tissue. In principle, one may envision a scenario in which editability itself is tissue-dependent: possibly, some of the determinants that characterize editability are not only due to the ability to bind ADARs but also related to the affinity to other competing RNA-binding proteins or RNA–RNA interactions (38). In different tissues, the effects of these affinities on the competition between the different RNA-binding protein could have different results due to variation in the relative levels of the proteins involved.

In addition, variability in transcript kinetics could result in different editing levels—faster splicing and transport out of the nucleus might lead to lower editing levels. Finally, some of the variability might have to do with cell-to-cell differences. Upcoming data from single cell sequencing might help to shed light on this possibility.
